# Granulocyte/Macrophage Colony-Stimulating Factor Influences Angiogenesis by Regulating the Coordinated Expression of VEGF and the Ang/Tie System

**DOI:** 10.1371/journal.pone.0092691

**Published:** 2014-03-21

**Authors:** Jingling Zhao, Lei Chen, Bin Shu, Jinming Tang, Lijun Zhang, Julin Xie, Shaohai Qi, Yingbin Xu

**Affiliations:** Department of Burns, the First Affiliated Hospital of Sun Yat-sen University, Guangzhou, China; Institute of Neurology (Edinger-Institute), Germany

## Abstract

Granulocyte/macrophage colony-stimulating factor (GM-CSF) can accelerate wound healing by promoting angiogenesis. The biological effects of GM-CSF in angiogenesis and the corresponding underlying molecular mechanisms, including in the early stages of primitive endothelial tubule formation and the later stages of new vessel maturation, have only been partially clarified. This study aimed to investigate the effects of GM-CSF on angiogenesis and its regulatory mechanisms. Employing a self-controlled model (Sprague-Dawley rats with deep partial-thickness burn wounds), we determined that GM-CSF can increase VEGF expression and decrease the expression ratio of Ang-1/Ang-2 and the phosphorylation of Tie-2 in the early stages of the wound healing process, which promotes the degradation of the basement membrane and the proliferation of endothelial cells. At later stages of wound healing, GM-CSF can increase the expression ratio of Ang-1/Ang-2 and the phosphorylation of Tie-2 and maintain a high VEGF expression level. Consequently, pericyte coverages were higher, and the basement membrane became more integrated in new blood vessels, which enhanced the barrier function of blood vessels. In summary, we report here that increased angiogenesis is associated with GM-CSF treatment, and we indicate that VEGF and the Ang/Tie system may act as angiogenic mediators of the healing effect of GM-CSF on burn wounds.

## Introduction

Wound healing involves a succession of complicated biochemical and cellular events, in which angiogenesis plays a significant role[1]. The formation time, quantity, and quality of new blood vessels in the wound can directly affect wound healing. In adults, the formation and maturation of new vessels are extremely complex and coordinated processes, requiring activation of a series of receptors and ligands to maintain balance between the stimulatory and inhibitory signals.

Vascular endothelial growth factor (VEGF) and the angiopoietin (Ang)/Tie-2 system are two types of vascular regulatory molecules that are crucial for vessel formation and maturation[2]. VEGF acts at early stages of angiogenesis, promoting the formation of primitive tubular structures that are prone to leakage and hemorrhage due to the single monolayer of endothelial cells. However, the Ang family, including Ang-1, Ang-2, and their receptor, tyrosine kinase Tie-2, exert functions at later stages of angiogenesis by mediating the interactions of endothelial cells and mural cells (pericytes)[3], promoting the maturation and stabilization of the new vessels due to the formation of endothelial tight junctions and the recruitment of pericytes[4].

Granulocyte macrophage colony-stimulating factor (GM-CSF) is a pleiotropic cytokine that can enhance the functions of various cells that are necessary for facilitating wound healing[5]. For example, GM-CSF activates monocytes/macrophages, promotes keratinocyte proliferation, and regulates the fibroblast phenotype[6]. GM-CSF has been shown to be secreted by keratinocytes in skin shortly after injury, which mediates epidermal cell proliferation in an autocrine manner[7]. Other cells which are the targets of GM-CSF involved in wound healing, including macrophages, fibroblasts, endothelial cells and dendritic cells, can also synthesize GM-CSF[8]. Animal and preclinical studies suggest that GM-CSF treatment accelerates wound healing[9], [10], and the factor has been applied in the treatment of human chronic skin wounds of different aetiology[11]. Mann et al. reported that wound repair was significantly enhanced in GM-CSF overexpression tg mice, with upregulation of the expression of some important cytokines[12]. Interestingly, researches showed that the exogenous application of GM-CSF has been proved to be effective in promoting wound repair, whereas, the intradermal administration of GM-CSF to the skin seem to can not accelerate cutaneous repair of acute wounds[13].

Despite its widely proven therapeutic effects, little is known about the molecular mechanisms underlying the action of GM-CSF in angiogenesis. Previous researches have suggested that GM-CSF applied locally can significantly increase the healing rate of wounds associated with vascular injury by increasing the formation of granulation tissue and enhancing angiogenesis[14], [15], which were mediated by elevating the expression of VEGF in the wound[16]. However, no previous studies have investigated the spatial and temporal patterns of Ang/Tie-2 system and VEGF expression in relation to angiogenesis during the different phases of wound healing after GM-CSF treatment.

In this study, we investigated the influence of GM-CSF on the entire process of angiogenesis in deep partial-thickness burn wounds in rats, including the early stage, involving formation of primitive endothelial tubules, and the later stage, involving maturation and stabilization of new blood vessels. Additionally, we discussed whether these effects could be mediated by the coordinated expression of Ang-1, Ang-2, Tie-2 and VEGF.

## Materials and Methods

### Animals

A total of 40 sex-matched Sprague-Dawley rats weighing 220–250 g were provided by the Experimental Animal Research Laboratory at Sun Yat-sen University in China. The animals were individually caged under specific pathogen-free (SPF) conditions with 12-hour light-dark cycles. They were allowed access to water and standard rat chow ad libitum and were monitored daily. Treatment of the animals was carried out in strict accordance with the recommendations described in the Guide for the Care and Use of Laboratory Animals of the National Institutes of Health. The protocol was approved by the Committee on the Ethics of Animal Experiments of Sun Yat-sen University. All operations were performed under chloral hydrate anesthesia, and all efforts were made to minimize suffering.

### Wound protocol

The burns were created using a 2.5-cm-diameter electrically heated brass rod as described previously with some modifications[17]. General anesthesia was provided via an intraperitoneal injection of 3 ml/kg chloral hydrate (10%). The backs of the rats were completely shaved with an electrical clipper. Deep partial-thickness burn wounds that were 0.5–1 cm off the midspinal line were made on each side of the dorsum of the rats by application of the electrically heated brass rod for 8 s with a constant pressure and temperature (1 kg, 80°C).

To prepare the GM-CSF solution, we mixed GM-CSF (PeproTech) with glycerin (10%) and mannitol (1%). Glycerin and mannitol functioned as protective agents to preserve the bioactivity of GM-CSF. Paired wounds on the same rat were randomly divided into the experimental group and the control group. GM-CSF solution (200 μl) was topically applied to the experimental group (the concentration of GM-CSF in the solution was 25 ng/μl, and the actual quantity of GM-CSF applied to the wound was 1 μg/cm^2^). The control group was treated with only protective agents as mentioned above (200 μl). All the wounds were covered with sterile gauze and bandaged. The experimental solutions were applied to the wounds daily, and the wounds were observed and assessed.

Eight rats were euthanized by cervical dislocation on the 1st, 3rd, 7th, 14th, and 21st days after being burned, and the full-thickness skin biopsies were harvested aseptically from the middle and periphery of the wounds.

### Wound analysis

The burn wounds on each rat were digitally photographed at the indicated time intervals, and the wound closure was assessed using Image-Pro Plus v. 6.0 (Media Cybernetics) under double-blind conditions. The wound areas were standardized by comparison with the original wound size, and the healing rate was expressed as a percentage of wound closure: [(day 0 area – day n area) / (day 0 area)] ×100% (n = 1, 3, 7, 14 or 21).

### Histology and immunohistology

Samples harvested from the wounds were fixed with 4% paraformaldehyde (PFA) and embedded in paraffin. Serial 5-μm sections were cut and stained with hematoxylin and eosin (HE) for observation of histological changes in the burn wounds.

For immunohistochemical staining, a panel of primary antibodies against rat Ang-1 (Santa Cruz Biotechnology, 1∶200), Ang-2 (Santa Cruz Biotechnology, 1∶150), Tie-2 (Santa Cruz Biotechnology, 1∶200), VEGF (Abcam, 1∶150), MMP-2 (Abcam, 1∶300), and MMP-9 (Abcam, 1∶200) were used. The activity of endogenous peroxidase was quenched by incubation with hydrogen peroxide (3%) for 5 min, followed by microware treatment at 500 W for 5 min in citrate buffer for antigen retrieval. Next, the sections were incubated with primary antibodies at 4°C overnight. After thorough washing, the sections were incubated in goat anti-rabbit antibody (Invitrogen, 1∶500) for 30 min, followed by incubation in avidin-biotin complex (Elite ABC kit; Vector Laboratories) for 30 min. Color was developed in 3′ 3-diaminobenzidine (Dako), and nuclei were stained with hematoxylin (Sigma). Negative control staining experiments were performed by omission of the primary antibody. Images were captured using a light microscope (Olympus BX51 WI).

### Immunofluorescence analysis

A double-labeling immunofluorescence technique was applied to analyze the proliferation of endothelial cells using anti-CD31 (Abcam, 1∶200) and anti-Ki67 (Abcam, 1∶600) antibodies. Similarly, the pericyte coverage of microvessels was detected using anti-CD31 and anti-α-smooth muscle actin (α-SMA) (Abcam, 1∶400) antibodies. The sections were blocked with BSA (5%) for 2 h and incubated with primary antibody at 4°C overnight. After thorough washing, the sections were further incubated with secondary goat anti-rabbit antibodies (Invitrogen, 1∶400) for 1 h in the dark. Subsequently, the sections were incubated in 4′6-diamidino-2 phenylindole (DAPI) for nuclear staining. Images were obtained with a fluorescence microscope (Carl Zeiss) and were merged using Image-Pro Plus v. 6.0 software. Negative control staining experiments were performed by omission of the primary antibody.

### Quantitation of the proliferating capillary index (PCI)

The PCI was used to assess the proliferating endothelial cells and was determined by calculating the ratio of the number of microvessels with proliferating endothelial cells (Ki67) to the total number of microvessels (CD31)[18]. The PCI was quantified in vascular hot spots that were identified by screening for the areas with the highest vessel density under a magnification of 200×.

### Quantification of microvessel density (MVD)

The MVD counting technique has been widely used to assess blood vessel number [19]. Here, CD31 was used as an endothelial cell marker, and CD31-positive endothelial cells and vessels with or without a lumen were counted under a power field of 10×20 in 5 randomly selected fields from 3 separate sections of each sample. The MVD was quantified as the average number of microvessels per viewing field.

### Quantification of the microvessel pericyte coverage index (MPI)

The MPI was used to assess the maturity of new blood vessels, and it was correspondingly established by quantifying the percentage of CD31-positive microvessels that showed colocalization of endothelial cell staining (CD31) and pericyte staining (a-SMA) under a power field of 10×20[18]. A single endothelial cell was regarded as a unit of quantification regardless of whether it formed a tube, and a pericyte was defined as a single layer of α-SMA-positive cells colocalizing with CD31-positive cells. For MPI quantification, at least five nonoverlapping microscopic fields per section were independently analyzed under double-blind conditions. The pericyte coverage was expressed as the α-SMA/CD31 ratio.

### Quantitative RT-PCR (qRT-PCR)

Total RNA was purified using the RNeasy Mini Kit (Qiagen) and was reverse-transcribed into cDNA using a thermocycler (S1000, Bio-Rad) and the First Strand cDNA Synthesis Kit (Fermentas) according to the manufacturer's protocol. The primer sequences were as follows: Ang-1 forward primer, GTC ACT GCA CAA AAG GGA CA, reverse primer, GGC TTA CAA GGA TGG CGT TA; Ang-2 forward primer, GTC TCC CAG CTG ACC AGT GGG, reverse primer, TAC CAC TTG ATA CCG TTG AAC; VEGF forward primer, CGA CAG AAG GGG AGC AGA AAG, reverse primer, GCA CTC CAG GGC TTC ATC ATT; MMP-2 forward primer, CAG GGA ATG AGT ACT GGG TCT ATT, reverse primer, ACT CCA GTT AAA GGC AGC ATC TAC; MMP-9 forward primer, AAT CTC TTC TAG AGA CTG GGA AGG AG, reverse primer, AGC TGA TTG ACT AAA GTA GCT GGA; and β-actin forward primer, CAG GTC ATC ACT ATC GGC AAT, reverse primer, GAG GTC TTT ACG GAT GTC AAC.

Real-time RT-PCR was performed using the SYBR qPCR mix (Toyobo) and a Real-Time PCR Detection System (Bio-Rad iQ5). The thermocycling profile for SYBR Green RT-PCR was as follows: an initial denaturation step at 95°C for 1 min, followed by 40 cycles of denaturation for 15 s at 95°C, annealing for 15 s at 60°C, and extension for 60 s at 72°C. Each sample was run in triplicate, and the relative gene expression was analyzed using the 2^−ΔΔCt^ method[20].

### Western blot analysis

The samples were homogenized, and total proteins were extracted using radioimmunoprecipitation assay (RIPA) buffer (Beyotime Biotechnology). A BCA kit (Beyotime Biotechnology) was then used to determine the protein concentrations. Different samples containing equal amounts of protein (60 μg) were separated on SDS polyacrylamide gels (10%), transferred to nitrocellulose membranes, and blocked in nonfat dry milk (5%) at room temperature for 2 h. The membranes were incubated overnight at 4°C with primary antibodies against Ang-1 (1∶1000), Ang-2 (1∶1000), Tie-2 (1∶1500), p-Tie-2 (Santa Cruz Biotechnology, 1∶500), VEGF (1∶2000), MMP-2 (1∶1500), MMP-9 (1∶1500), and β-actin (1∶5000, Bioworld Technology). Finally, the membranes were incubated with horseradish peroxidase-conjugated secondary antibody and detected using an enhanced chemiluminescence substrate (Beyotime Biotechnology).

### Transmission electron microscopy

The sections were fixed in glutaraldehyde (2.5%) and osmium tetroxide (1.0%) for 24 and 1.5 h, respectively, and were embedded in Epon812 after being dehydrated in a graded ethanol series. Ultrathin sections were cut on an ultramicrotome (LKB-V) and were mounted on Formvar-coated slit grids. Ultrathin sections were stained with uranyl acetate and lead citrate for observation using a transmission electron microscope (Hitachi H-500).

### Vascular permeability assay

Vascular permeability was quantified according to a previously described method [21] by intravenous administration of Evans blue dye (Sigma) and examination of its diffusion into the skin[22]. Evans blue dye dissolved in dimethylformamide (DMF, 0.5%, Sigma) was injected through the caudal vein at a dose of 18 mg/kg. At 40 min after injection, the rats were perfused via the left ventricle with 500 ml of pre-warmed (37°C) PBS to remove the intravascular dye. Then, the skin at a distance of 0.5 cm from the wound was dissected and weighed, and the Evans blue dye was extracted by incubation in DMF at 65°C overnight. The extract was centrifuged at 3000 rpm for 15 min and measured with a spectrophotometer (Nanodrop 2000). The concentration of Evans blue in the extracts was calculated from a standard curve of Evans blue in DMF, and the vascular permeability rate was expressed as the percentage of Evans blue content in the skin samples (μg/kg).

### Statistical analyses

Comparisons of the healing rate, proliferating capillary index, microvessel pericyte coverage index, number of pericytes, microvascular density, changes in levels of mRNA and protein, and the vascular permeability between control and GM-CSF groups at the same time point were conducted using Student's t test. The differences between the groups at different time points were compared by one-way ANOVA, followed by the Bonferroni test. All statistical analyses were performed using SPSS 18.0 software (SPSS, Chicago, IL, USA), with P<0.05 considered to be statistically significant.

## Results

### Wound closure

**Figure 1:**
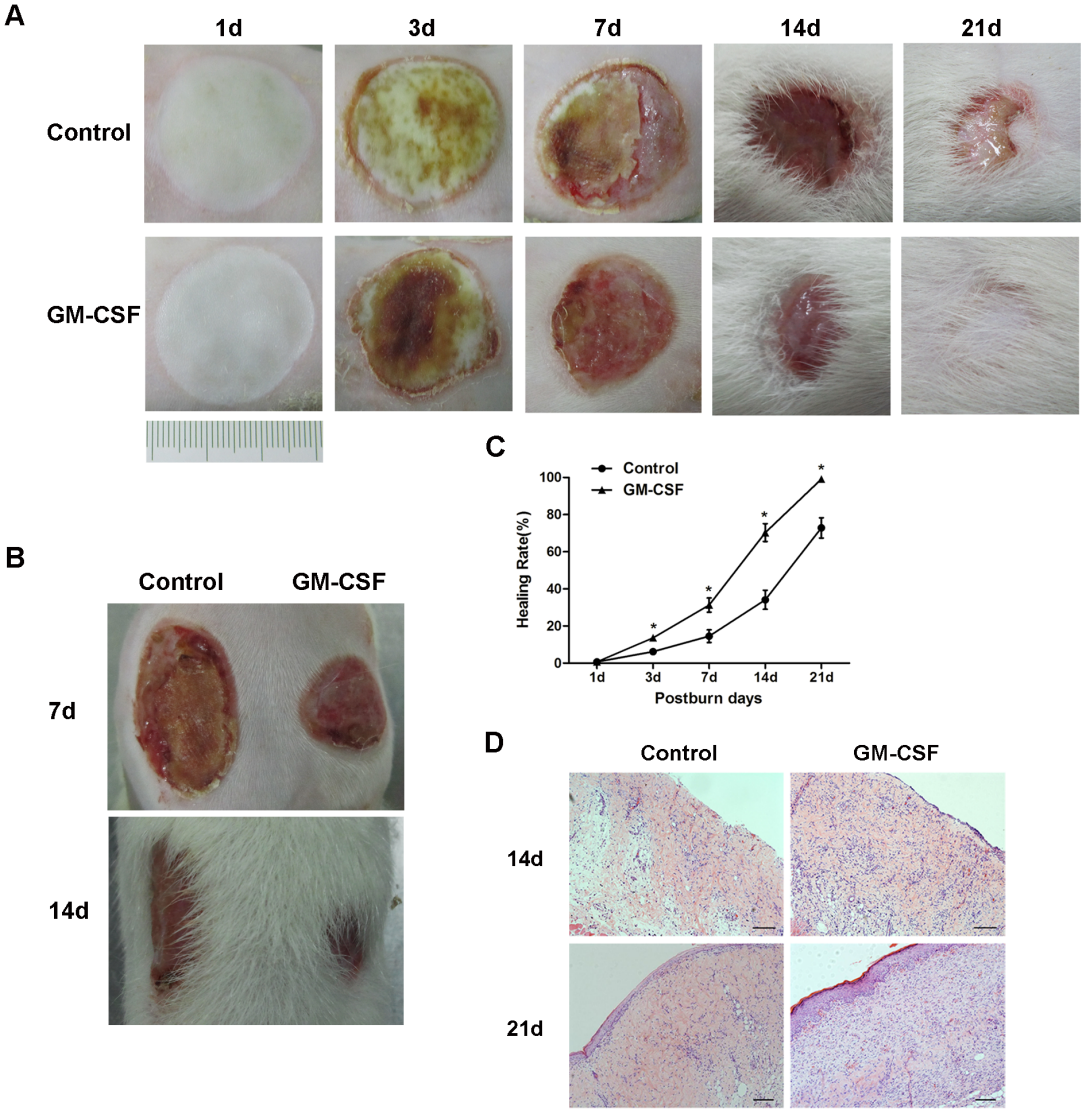
The speed and quality of burn wound healing. **(A)** Photographs showing macroscopic wound healing in both the control and GM-CSF treatment groups at different time points following the burn event. **(B)** Wound healing in the control and GM-CSF treatment groups in self-controlled rat models at days 7 and 14 after the burn event. **(C)** Graph showing the wound healing rate in the two groups (mean±SD) (*P<0.05). **(D)** Histological changes in burn wounds in the two groups at days 14 and 21 after the burn event. Scale bar = 50 mm.

Wound closure progressed quickly in the experimental group; the crusta on the wound margins was separated on the 3rd day after burning and fell off completely on the 14th day, when the healing area was more than half of the original area. By the 21st day, the wound reached complete closure (Fig. 1A). In contrast, the decrustation and epithelization of the wound in the control group was significantly delayed compared with that in the experimental group (Fig. 1B), and the wound healing rate was much slower than that in experimental group, particularly on the 3rd, 7th, 14th, and 21st post-burn days (P<0.05) (Fig. 1C).

### Histopathological assessments

In the early stages of wound healing, the degrees of epidermal necrolysis and inflammatory cell infiltration were more severe in the control group than in the experimental group. In the late stages of wound healing in the experimental group, the new blood vessels were abundant, the epidermal layer was thicker during re-epithelialization, the collagen fibrils were distributed compactly and regularly, and the accessory structures, such as hair follicles in the dermis, were obviously regenerated. However, the number of new blood vessels was less and the epidermal layer was thinner in the control group; additionally, the collagen fibrils were broken, and the accessory structures were destroyed or missing (Fig. 1D).

### Endothelial cell proliferation in burn wounds

**Figure 2:**
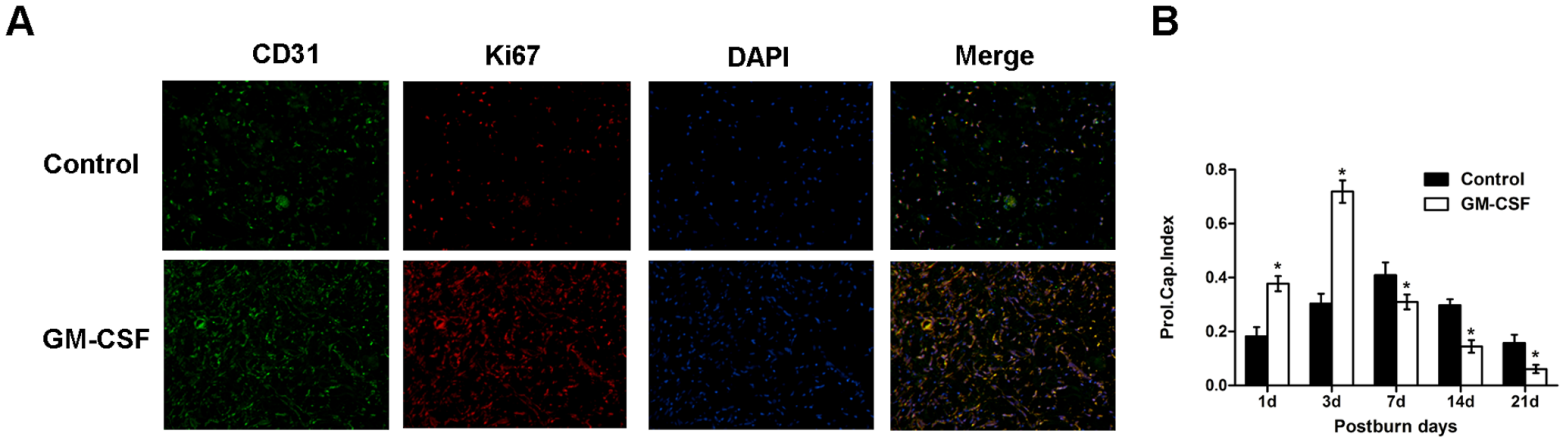
Immunofluorescence assay of proliferating endothelial cells in the wound. **(A)** Representative examples of double staining of Ki67/CD31 (green, CD31; red, Ki67; nucleus, blue) in skin sections from the control group and the GM-CSF treatment group at day 3 after the burn event. Original magnification, ×200. (B) Quantitative comparison of the proliferating capillary index (PCI) in the two groups. The PCI was used to assess the percentage of microvessels with proliferating endothelial cells. All data are expressed as the mean±SD (*P<0.05).

To quantify endothelial cell proliferation in the wounds, we simultaneously stained endothelial cells to determine the expression of CD31, and we identified proliferating cells using the proliferation marker Ki67 (Fig. 2A). When quantifying the PCI, which is reflected by the percentage of microvessels with Ki67-positive endothelial cell nuclei, significant differences were detected between the two groups. As shown in Fig. 2B, the PCI values increased sharply from the 1st to the 3rd day, and the experimental group had significantly higher PCI values than the control group during that time (P<0.05). Then, the PCI values decreased from the 7th to the 21st day, and they were lower in the experimental group than in the control group (P<0.05).

### Microvessel density and pericyte coverage of microvessels

**Figure 3:**
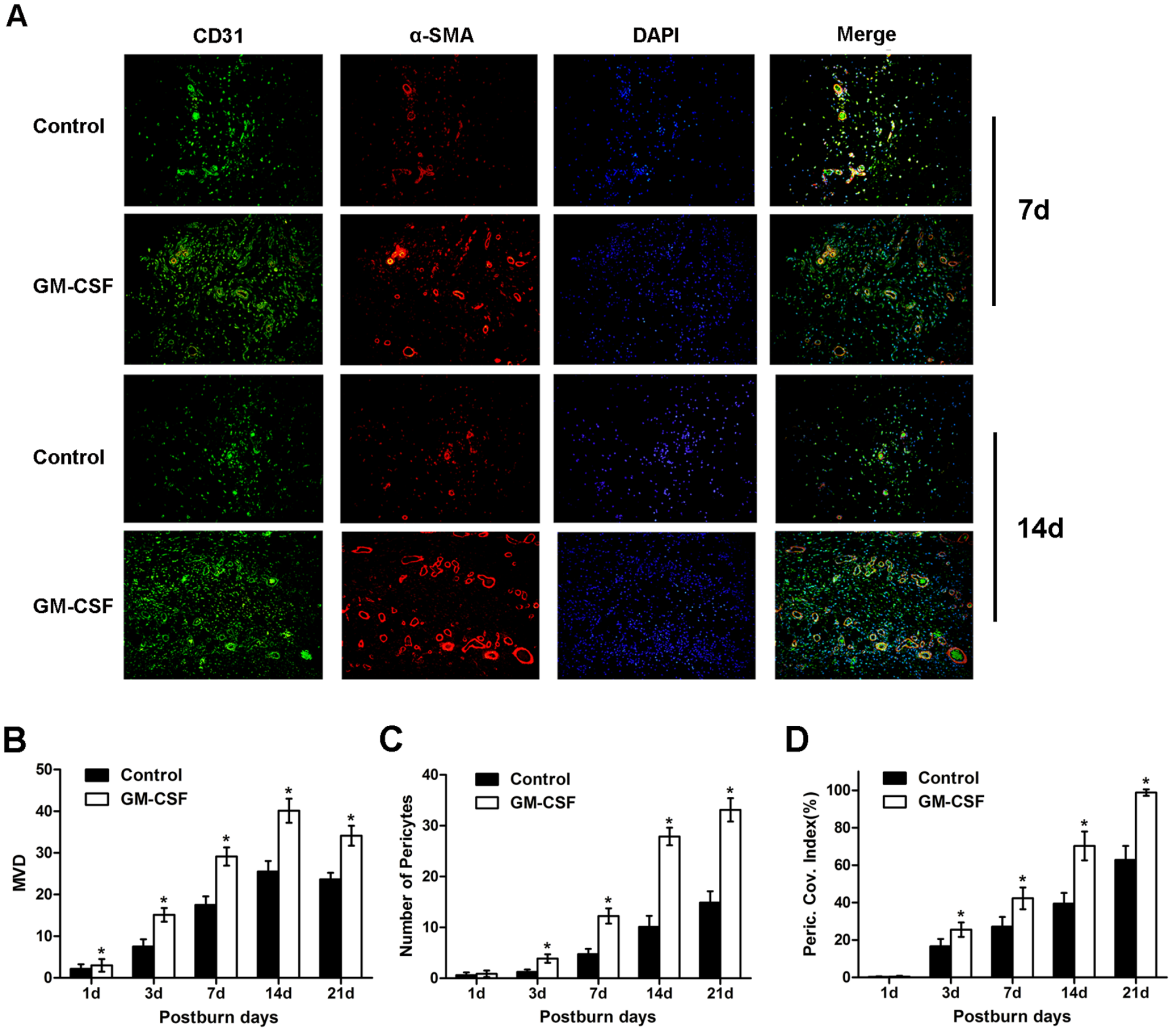
Immunofluorescence analysis of microvascular density (MVD) and pericyte coverage of microvessels in the wound. **(A)** A double-labeling technique was used to stain endothelial cells for CD31 expression (green) and pericytes for α-SMA expression (red). Representative images of the 2 groups at days 7 and 14 after the burn event are shown. Original magnification, ×200. **(B)** The MVD in the two groups at different time intervals. **(C)** The number of pericytes in the two groups at different time intervals. **(D)** The microvessel pericyte coverage index (MPI) was quantified by assessing the percentage of microvessels that were associated with α-SMA-positive pericytes. All data are expressed as the mean±SD (*P<0.05).

We used a combination of the specific markers CD31 and α-SMA to simultaneously immunostain vascular endothelial cells and pericytes to quantitatively assess the functional status of the neovasculature in the wounds[23] (Fig. 3A). The results showed that there were a small number of CD31-positive cells in both groups on the 1st day. Then, some green-stained CD31-positive endothelial cells could be detected on the 3rd day, and the number of endothelial cells was obviously increased beginning on the 7th day and peaked on the 14th day; it then decreased slightly on the 21st day. The MVD was used to quantify the number of microvessels, and the results showed that the experimental group had much higher average MVDs than the control group from the 3rd to the 21st days post-burn (P<0.05) (Fig. 3B). Meanwhile, when we observed the red-stained α-SMA-positive pericytes, we found that the pericytes appeared in small numbers in both groups on the 3rd day. Subsequently, pericytes could be more easily detected and covered the endothelial cells discontinuously on the 7th day. Then, the pericytes formed circles and densely enveloped the endothelial cells on the 14th day in the experimental group. Until the 21st day, almost every microvessel colocalized with pericytes in the experimental group, whereas only some of the microvessels were covered with pericytes in the control group. When comparing the number of pericytes between the two groups, the results showed that the number of pericytes was higher in the experimental group than the control group from the 3rd to the 21st day post-burn (P<0.05) (Fig. 3C).

Although the MVD values could be used to assess the presence of blood vessels, these values did not provide an indication of the maturity of the neovasculature or the functional status of the vessels[18]. Therefore, the MPI was used to reflect the percentage of microvessels covered with pericytes and was quantified to evaluate the maturity of the neovasculature. As shown in Fig. 3D, the MPI was 0 on the 1st day in both groups, and it increased from the 3rd to the 21st day post-burn. The MPI in the experimental group was higher than that in the control group at the above time points (P<0.05).

### Expression changes in Ang-1, Ang-2, and Ang-1/Ang-2 after GM-CSF treatment

**Figure 4:**
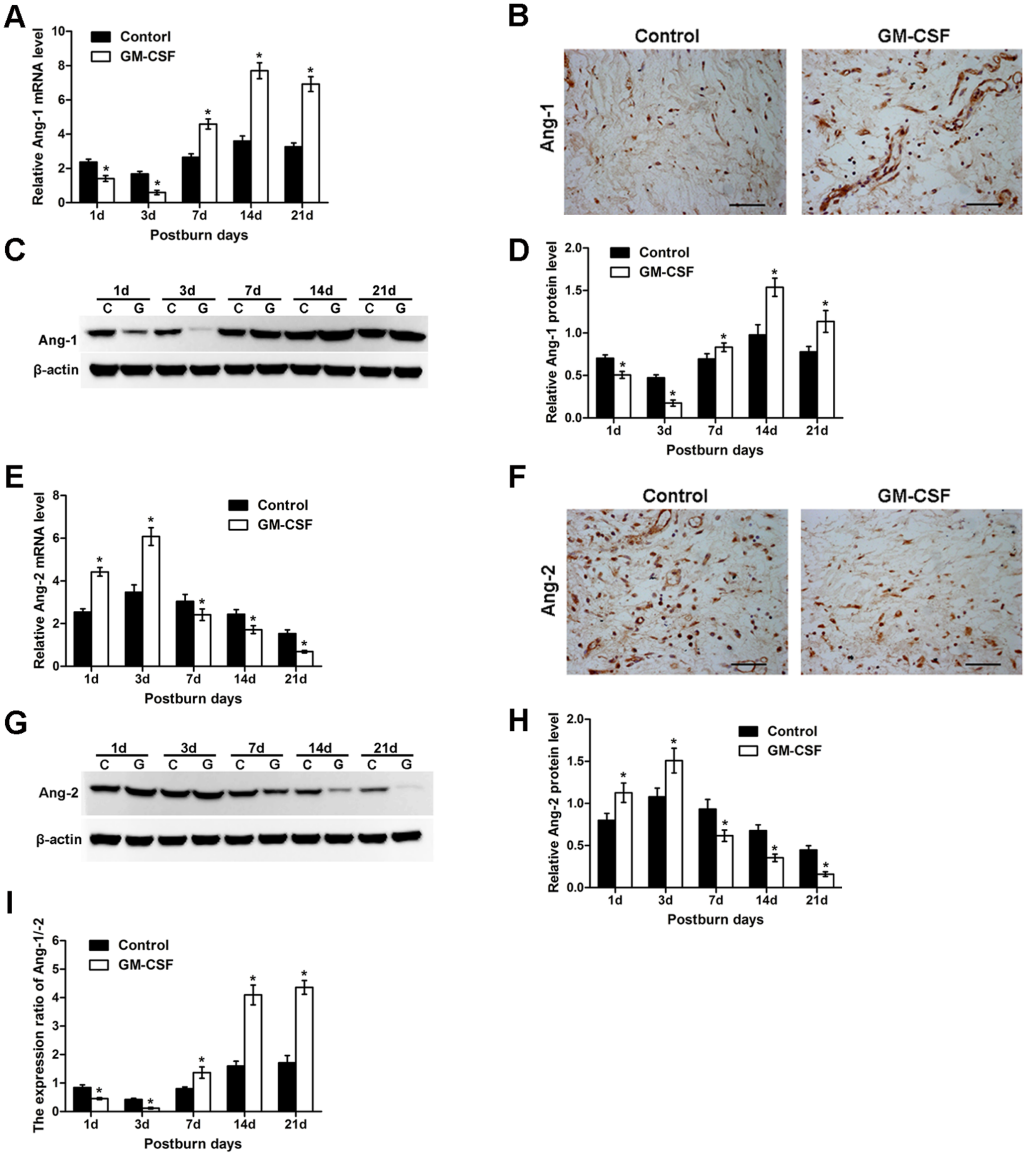
Expression of Ang-1 and Ang-2 in the skin during wound healing. **(A)** Ang-1 mRNA levels in the control and GM-CSF treatment groups were assessed by qRT-PCR. **(B)** Immunohistochemical staining of Ang-1 protein in the two groups at day 7 after the burn event. Ang-1 protein signals were present mainly in pericyte-like perivascular mural cells. Scale bar = 100 mm. **(C)** Representative Western blots showing Ang-1 protein levels in the two groups during the healing process. **(D)** Statistical analysis of Ang-1 protein levels. **(E)** qRT-PCR analysis of Ang-2 mRNA expression levels in the two groups. **(F)** Immunohistochemical staining of Ang-2 protein at day 7 after the burn event. Ang-2 protein signals were present in capillary luminal and perivascular mural cells. Scale bar = 100 mm. **(G)** Representative Western blots showing Ang-2 protein levels during the healing process. **(H)** Statistical analysis of Ang-2 protein levels. **(I)** The protein expression ratio of Ang-1/Ang-2. All data above are expressed as the mean±SD (*P<0.05).

The changes in gene expression of Ang-1 after GM-CSF treatment were quantitatively analyzed by qRT-PCR. As shown in Fig. 4A, the mRNA expression of Ang-1 was decreased on the 1st and 3rd days post-burn, and that in the experimental group was significantly lower than that in the control group (P<0.05). Then, Ang-1 mRNA increased gradually on the 7th day and peaked on the 14th day before decreasing slightly on the 21st day, and its expression level in the experimental group was much higher than that in the control group at the above time points (P<0.05). By immunohistochemical analysis, we found that Ang-1 was mainly expressed in pericyte-like perivascular mural cells, and Ang-1-positive staining in the experimental group was higher than that in the control group on the 7th day (Fig. 4B). Representative Western blots of Ang-1 protein expression at different time points are shown in Fig. 4C, which presented the same trend as Ang-1 mRNA expression in the two groups. The results of the statistical analysis of Ang-1 protein expression are shown in Fig. 4D.

Changes in the mRNA expression of Ang-2 are shown in Fig. 4E. The results suggested that Ang-2 mRNA expression was elevated on the 1st and 3rd days and that it was significantly higher in the experimental group compared with the control group (P<0.05). Then, Ang-2 expression was reduced sharply from the 7th day and was maintained at a low level until the 21st day; the expression levels on these days were significantly lower in the experimental group compared with the control group (P<0.05). Immunohistochemical staining revealed that Ang-2 was present in the endothelial cell- and pericyte-like capillary walls, and the Ang-2-positive staining in the experimental group was decreased compared with that in the control group on the 7th day (Fig. 4F). The change in Ang-2 protein expression (Fig. 4G–H) was consistent with its mRNA expression pattern.

When calculating the Ang-1/Ang-2 expression ratio (Fig. 4I), we found that in the experimental group, the ratio was much lower than that in the control group on the 1st and 3rd days. This ratio was notably higher in the experimental group on the 7th, 14th, and 21st days compared with that in the control group (P<0.05).

### Expression changes in pTie-2 and VEGF after GM-CSF treatment

**Figure 5:**
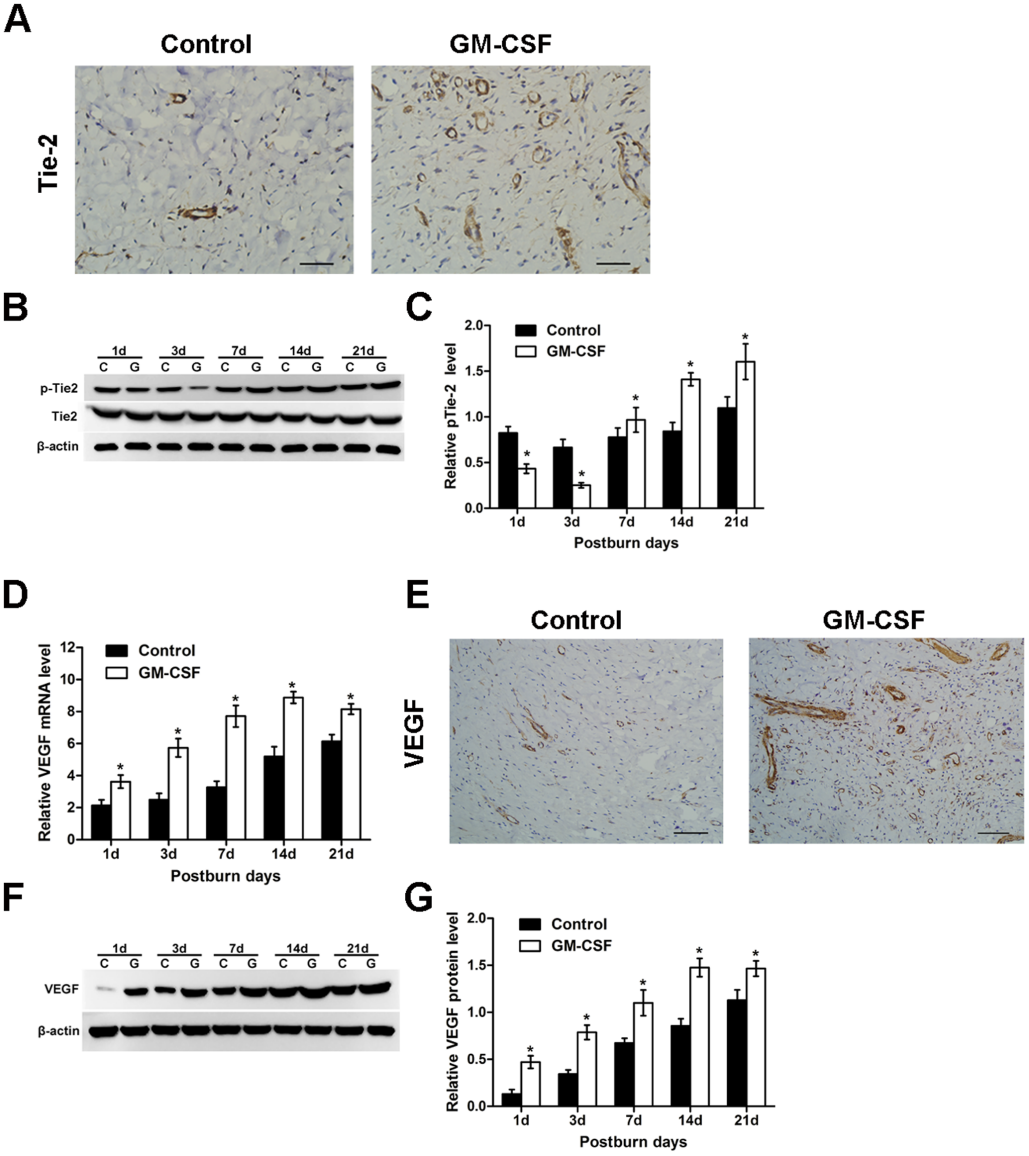
Tie2 protein levels, tyrosine phosphorylation, and VEGF expression in the skin wounds. **(A)** Immunohistochemical staining of Tie-2 protein in the two groups at day 7 after the burn event. Tie-2 protein signals were present mainly in endothelial cells. Scale bar = 100 mm. **(B)** Representative Western blots showing Tie2 tyrosine phosphorylation levels and Tie2 protein levels in the two groups. **(C)** Quantification of the relative intensity of phosphotyrosine-Tie-2 compared with total Tie-2. **(D)** qRT-PCR analysis of VEGF mRNA expression levels in the two groups. **(E)** Immunohistochemical staining of VEGF protein at day 7 after the burn event. VEGF protein signals were present in pericyte-like perivascular mural cells. Scale bar = 100 mm. **(F)** Representative Western blots showing VEGF protein levels during the healing process. **(G)** Statistical analysis of VEGF protein levels. All of the above data are expressed as the mean±SD (*P<0.05).

Immunohistochemical staining revealed that Tie-2 was mainly expressed in the endothelial cells (Fig. 5A). The tyrosine phosphorylation of Tie-2 was detected by Western blot analysis. As shown in Fig. 5B and C, the expression of pTie-2 was reduced from the 1st to the 3rd day and subsequently increased from the 7th to the 21st day. Compared with that in the control group, the expression of pTie2 in the experimental group was much lower on the 1st and 3rd days and was significantly higher on the 7th, 14th, and 21st days (P<0.05). The results showed that VEGF expression was increased immediately after the burn event and showed a tendency to increase until the 21st day. As shown in Fig. 5D, the mRNA expression of VEGF in the experimental group was significantly higher on the 1st, 3rd, 7th, 14th, and 21st days than that in the control group (P<0.05). By immunohistochemical analysis, we found that VEGF was mainly expressed in pericyte-like perivascular mural cells (Fig. 5E), and its protein expression in the experimental group was much higher than that in the control group at different time points (Fig. 5J and K).

### The expression of matrix metalloproteinase-2 (MMP-2) and MMP-9

**Figure 6:**
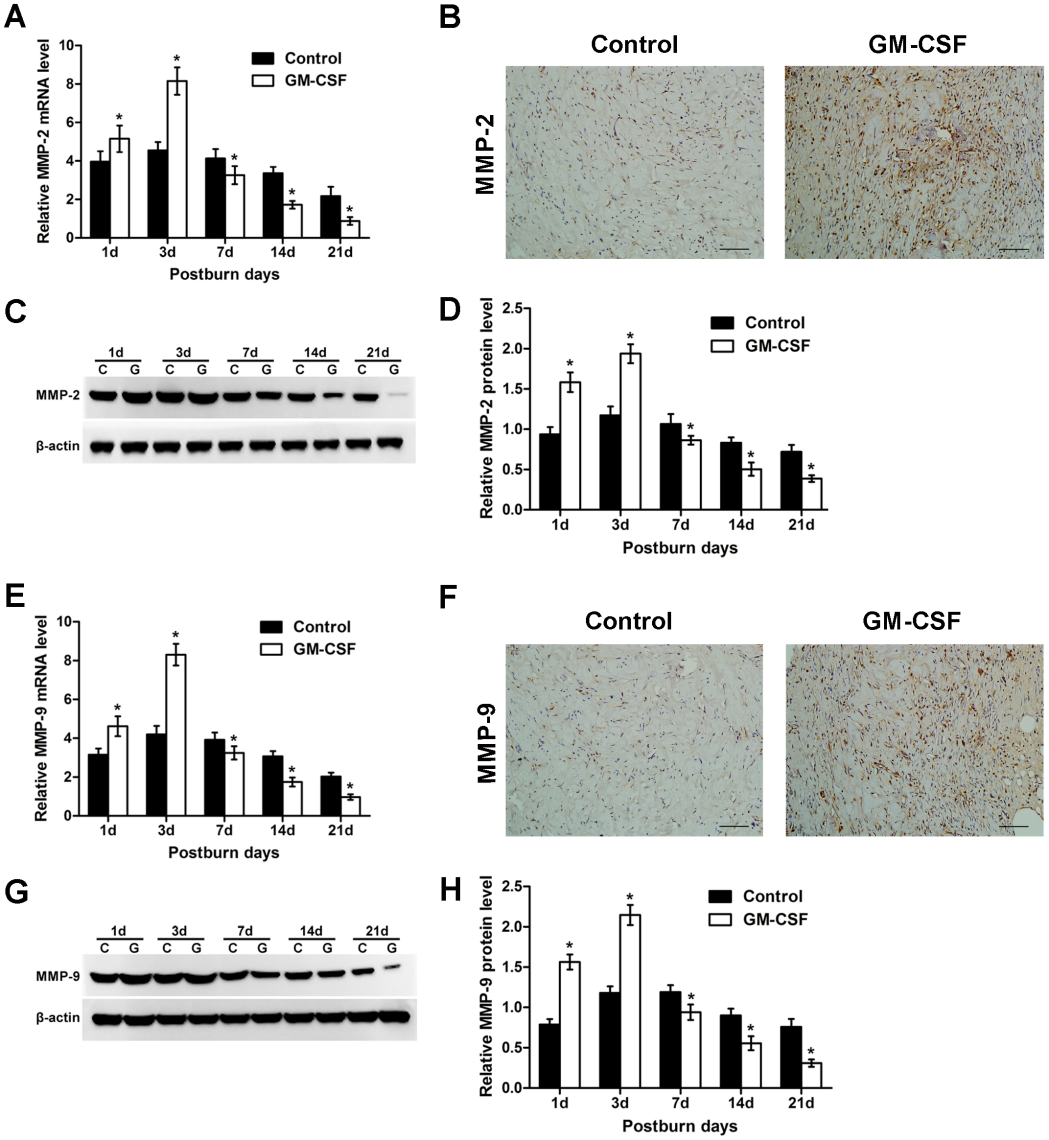
Time course of changes in MMP-2 and MMP-9 expression in the wounds. **(A)** MMP-2 mRNA levels in the control and GM-CSF treatment groups were assessed by qRT-PCR. **(B)** Immunohistochemical staining of MMP-2 protein in the two groups at day 3 after the burn event. Scale bar = 100 mm. **(C)** Representative Western blots showing MMP-2 protein levels in the two groups during the healing process. **(D)** Statistical analysis of MMP-2 protein levels. **(E)** qRT-PCR analysis of MMP-9 mRNA expression levels in the two groups. **(F)** Immunohistochemical staining for MMP-9 protein at day 3 after the burn event. Scale bar = 100 mm. **(G)** Representative Western blots showing MMP-9 protein levels during the healing process. **(H)** Statistical analysis of MMP-9 protein levels. All data above are expressed as the mean±SD (*P<0.05).

A critical event during the process of angiogenesis is the proteolytic breakdown of the basement membrane and migration of endothelial cells through the extracellular matrix (ECM)[24]. This process involves secretion and activation of MMP-2 and MMP-9, which are secreted by activated endothelial cells and exert functions during the initial stage of angiogenesis[25]. As shown in Fig. 6A, the mRNA expression of MMP-2 in the experimental group increased markedly on the 1st and 3rd days and was much higher than that in the control group (P<0.05). Then, the expression of MMP-2 gradually decreased from the 7th day and was maintained at a low level until the 21st day; the expression level in experimental group was obviously lower than that in the control group on these days (P<0.05). Fig. 6B shows the immunohistochemical staining of MMP-2 in the burn wounds, and the protein expression of MMP-2 is shown in Fig. 6C. Protein expression presented the same trend as mRNA expression in the two groups (Fig. 6D). As shown in Fig. 6E and H, we found that the trends of MMP-9 mRNA and protein expression in the two groups were the same as those of MMP-2, and there were significant differences between the two groups.

### Ultrastructures and anti-leakage functions of microvessels

**Figure 7:**
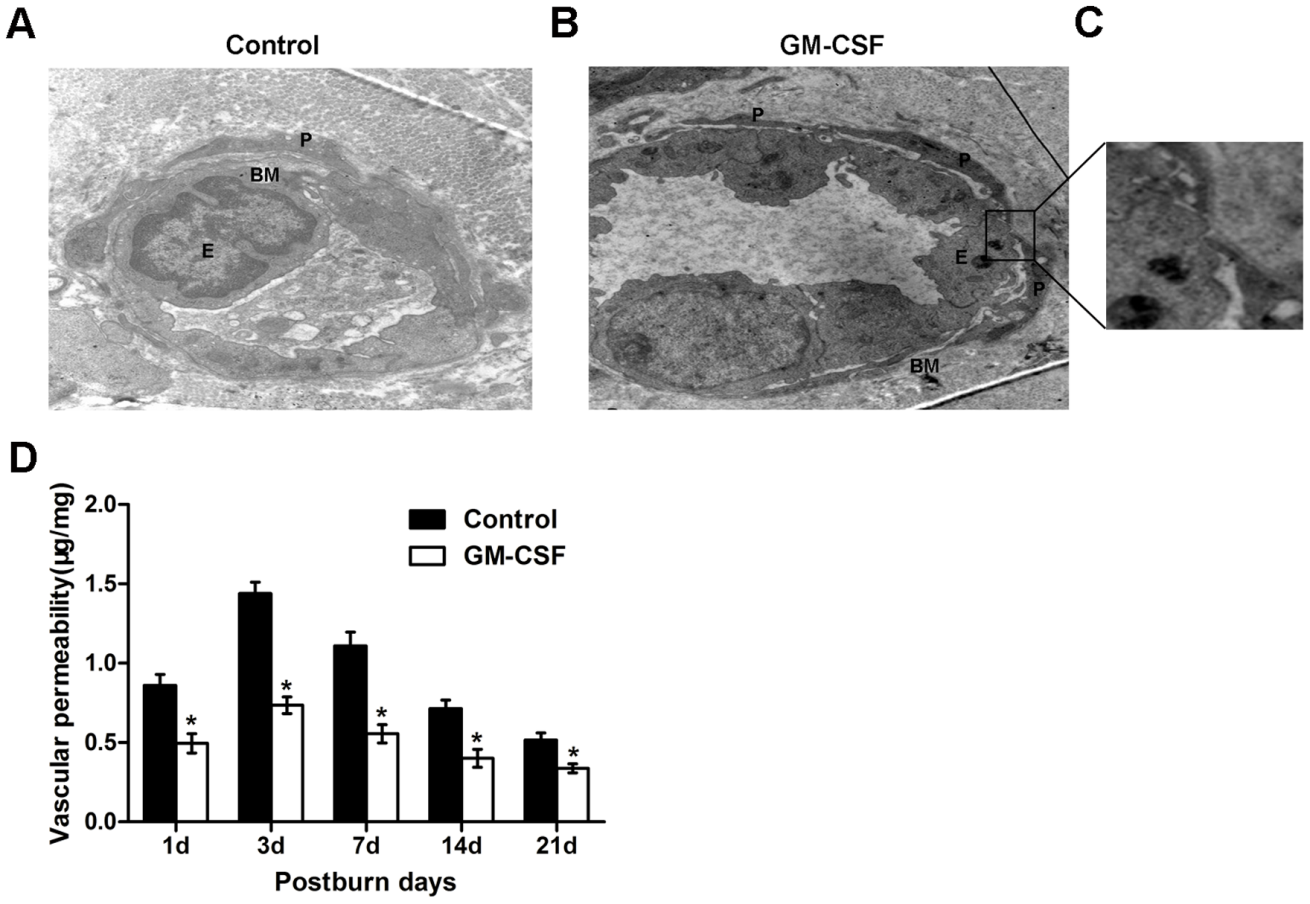
Electron micrographs of the newly formed microvessels in the wounds. **(A)** Ultrastructural characterization of microvessels in the control group at day 14 after the burn event. The microvessels have a poorly organized basement membrane and swelled endothelial cells. Few pericytes coated the endothelium. Original magnification, ×5800. **(B)** In the GM-CSF treatment group, the endothelial cells formed tight junctions, several elongated pericytes were embedded within an integrated basement membrane, and their longitudinal processes completely surrounded the endothelial tube. E, endothelial cell; P, pericyte; BM, basement membrane. Original magnification, ×5800. **(C)** Enlargement of the image in the black box. A pericyte can be observed making direct contact with an endothelial cell. A high-power view of an EPI is shown, which is composed of a pericyte cytoplasmic projection and corresponding endothelial indentation. **(D)** Time course of vascular permeability changes after the burn event in the two groups. Data are expressed as the mean±SD (*P<0.05).

A transmission electron microscope was used to observe the ultrastructural features of microvessels in the burn wounds. As shown in Fig. 7A, the structure of microvessels was abnormal in the control group. The lumen was narrowed by swelling endothelial cells, in which agglutinative chromatin and degenerated mitochondria were observed. Compared with the experimental group, fewer pericytes with larger interspaces were located around the endothelial cells, and the basement membrane was obviously thickened and broken in the control group. However, in the experimental group, the cross-sectional shapes of microvessels were round or ellipsoidal, and the endothelial cells were arranged in a monolayer to form tight junctions. Several pericytes with elongated, stellate shapes enveloped vessels continuously and tightly, and these pericytes shared a clear and integral basement membrane with endothelial cells. Furthermore, many endothelial cells and pericyte interdigitations (EPIs) could be detected in the experimental group (Fig. 7B). Fig. 7C shows a magnified image of an EPI in the experimental group. EPIs that were observed in angiogenic sites were defined as ultrastructural endothelial cells and pericyte cytoplasmic interdigitations[26], which may be a mechanical anchoring system for the two cell types[27] to help maintain the stability of microvessels. Fig. 7D shows a time course analysis of vascular permeability in the burn wounds. Here, we used the transfer of Evans blue dye from the plasma into the skin as a marker with which to evaluate the changes in vascular permeability in the burn wounds, which can indirectly reflect the vascular barrier function. The results showed that the vascular permeability began to elevate on the 1st day and reached a peak on the 3rd day after the burn event; it then significantly decreased on the 7th and 14th days and decreased slightly on the 21st day, when the permeability rate was close to that of the normal skin. Compared with that in the control group, the permeability rate was lower in the experimental group at each of the time points above (P<0.05).

## Discussion

GM-CSF is a highly pleiotropic cytokine with many diverse biologic effects such as stimulating proliferation and differentiation of hematopoietic progenitor cells into eosinophils, macrophages, and neutrophils[28], [29]. Therapeutically, GM-CSF is primarily used in patients with hematopathy to improve neutrophil recovery and mobilize peripheral-blood progenitor cells[30]. In recent years, it has been demonstrated that apart from accelerating hemopoietic recovery, GM-CSF also can influence the biologic activities of several cells that are essential in the wound repair[31]. In some researches, GM-CSF has been employed in the treatment of poorly healing wounds of diverse etiologies with some success[32], [33]. Consistent with these observations, we found that the speed as well as the quality of burn wound healing were improved after GM-CSF treatment. Researches on deep partial-thickness burn wound showed that microvascular injury followed by thermal injury-induced local microcirculation dysfunction of the skin is a major reason for delayed wound healing. Therefore, promoting angiogenesis is an effective strategy to cure the burn wound. Increased angiogenesis has been reported in association with accelerated wound healing in transgenic mice overexpressing GM-CSF in the skin. Other studies also showed that GM-CSF can facilitate wound reepithelialization by accelerating neovascularization[5]. However, they didn't discuss whether the new vessels are fully mature and functional. In the present study, we found that topical application of GM-CSF can not only increase the microvascular density, but also promote the maturation and stabilization of microvessels in the wound.

To further understand how GM-CSF mediated the whole process of angiogenesis, including the formation and maturation of new microvessels, we examined the corresponding expression of VEGF and the Ang/Tie system, which can regulate the emergence and maturation of new vessels, respectively, during wound healing[34] . Previous researches showed that increased microvascular density resulting from GM-CSF treatment is associated with the expression of the proangiogenic factor VEGF[11], [33], without mentioning the expression changes of the Ang/Tie system and the functional status of the neovasculature. The Ang family of growth factors includes Ang-1, Ang-2, mouse Ang-3, and human Ang-4, all of which bind to the endothelial receptor tyrosine kinase Tie-2. Ang-1 and Ang-2, the first two members of Ang family which have been most extensively studied, are considered important regulators of vascular remodeling and maturation[35]. However, mouse Ang-3 and human Ang-4 are interspecies orthologues and represent the third member of the Ang family[36]. Ang-3 is an agonist for the Tie-2 receptor in mouse endothelium and Ang-4, which expressed specially in human lung tissue, is identified as an agonist of Tie-2[37]. Based on these findings, we detected the expression of Ang-1, Ang-2 and Tie-2, the important regulatory factors in angiogenesis during the wound healing. We found that in the early stage of burn wound healing (1–3 days), the expression of Ang-1 and p-Tie-2 were reduced, whereas the expression of Ang-2 and VEGF were increased after GM-CSF treatment. It has been suggested that Ang-1 is a pericyte-derived microvessel stabilizing factor, which can bind to the Tie-2 receptor, which is predominantly expressed on endothelial cells, and activate Tie-2 by inducing its phosphorylation[38]. Constitutive Ang-1 expression and Tie-2 phosphorylation mediate the maturation and quiescence of the microvascular endothelium[3], [39], [40]. In contrast, Ang-2 serves as an antagonist and inhibits Ang-1-induced phosphorylation of Tie-2 in endothelial cells[41], [42]. By blocking the stabilizing action of Ang-1/Tie-2 signaling, Ang-2 initiates extensive angiogenesis, such as pericyte drop-off and extracellular matrix alteration[43], [44], which can facilitate microvessel sprouting. However, the effect of Ang2 on angiogenesis depends on the presence of VEGF. Microvessels would degenerate if VEGF was not expressed[45]. In this study, we found that in addition to maintaining the elevated level of VEGF that promotes the proliferation of endothelial cells, GM-CSF can also reduce the Ang-1/Ang-2 expression ratio in the initial phase of microvessel sprouting, which results in high-level expression of MMP-2 and MMP-9. As MMP-2 and MMP-9 function in extracellular proteolysis[46] and are responsible for facilitating endothelial cell migration[25] by degrading the extracellular matrix (ECM), the high-level expression of MMP-2 and MMP-9 suggesting that the sprouting phase of angiogenesis progresses rapidly after GM-CSF treatment.

After the formation of primitive endothelial tubules, the stabilization and maturation of the newly formed vessels become the main focus. Some studies emphasized that the maturation of vessels is vital because insufficient maturity of the blood-vascular system may lead to vascular occlusion, edema, and hemorrhage, which block transport of the nutrients and oxygen required for wound closure[47]. In this process, Ang-1, the microvessel-stabilizing factor, plays an important role by promoting the recruitment of pericytes[48]. Studies demonstrated that pericytes make direct intercellular contacts with endothelial cells and share the basement membrane with them[49]. In addition, pericytes and the basement membrane serve as important vascular support structures that maintain the stability of endothelial cells[50]. Therefore, the coverage of pericytes and integrity of the basement membrane are main characteristics of the mature microvessels. When we observed the newly formed microvessels in the later stage of wound healing (7–21 days), we found that with the high expression ratio of Ang-1/Ang-2 and the high level of Tie2 phosphorylation after GM-CSF treatment, the microvessels had a high coverage ratio of pericytes. Several elongated pericytes enveloped endothelial cells and bridged neighboring endothelial cells to form tight junctions, indicating that these microvessels were much more mature and stable, and the function of GM-CSF to facilitate microvascular maturation was associated with the high expression of Ang-1. Furthermore, Ang-1 was shown to be effective in inhibition of the high permeability of microvessels induced by VEGF through altering the phosphorylation of VE-cadherin and PECAM-1[51]. Therefore, it can be speculated that GM-CSF aids in the formation of leakage-resistant microvessels. We confirmed this speculation using the Evans Blue leakage method, which showed that GM-CSF can significantly reduce vascular permeability, indicating that the barrier functions of new microvessels could be improved after GM-CSF treatment.

In summary, GM-CSF is an angiogenic factor that can accelerate burn wound healing by promoting the vascularization process. Our study demonstrates that in addition to regulating the expression of VEGF in the wound, GM-CSF can also mediate the Ang-1/Ang-2 expression ratio and the phosphorylation of Tie-2. Thus, GM-CSF not only initiates the sprouting phase of angiogenesis but can also promote the maturation and stabilization of new microvessels. The results of this study provide a strong rationale for the exploration of GM-CSF as a therapeutic strategy for targeting diseases related to disorders of mature microvessels by manipulating the spatial-temporal Ang-1/Ang-2 balance.
